# Chloroquine synergizes with FTS to enhance cell growth inhibition and cell death

**DOI:** 10.18632/oncotarget.1500

**Published:** 2013-11-20

**Authors:** Eran Schmukler, Eya Wolfson, Roni Haklai, Galit Elad-Sfadia, Yoel Kloog, Ronit Pinkas-Kramarski

**Affiliations:** ^1^ Department of Neurobiology. Tel-Aviv University, Ramat-Aviv, Israel.

**Keywords:** Autophagy, Ras, Transformation, signal transduction

## Abstract

The Ras family of small GTPases transmits extracellular signals that regulate cell growth, differentiation, motility and death. Ras signaling is constitutively active in a large number of human cancers. Ras can also regulate autophagy by affecting several signaling pathways including the mTOR pathway. Autophagy is a process that regulates the balance between protein synthesis and protein degradation. It is important for normal growth control, but may be defective in diseases. Previously, we have shown that Ras inhibition by FTS induces autophagy, which partially protects cancer cells and may limit the use of FTS as an anti-cancer drug. Since FTS is a non toxic drug we hypothesized that FTS and chloroquine (an autophagy inhibitor) will synergize in cell growth inhibition and cell death. Thus, in the present study, we explored the mechanism of each individual drug and their combined action. Our results demonstrate that in HCT-116 and in Panc-1 cells, FTS induces autophagy, which can be inhibited by chloroquine. Furthermore, the combined treatment synergistically decreased the number of viable cells. Interestingly, the combined treatment enhanced apoptotic cell death as indicated by increased sub-G1 cell population, increased Hoechst staining, activation of caspase 3, decrease in survivin expression and release of cytochrome c. Thus, chloroquine treatment may promote FTS-mediated inhibition of tumor cell growth and may stimulate apoptotic cell death.

## INTRODUCTION

The Ras family of small GTPases transmits signals initiated by cell surface receptors, to regulate several cellular processes such as cell growth, differentiation, motility and death [1]. Activated Ras transmits signals through its interaction with multiple effectors, including mitogen activated kinase (MAPK), phosphoinositide-3-kinase (PI3K) and Ral-GEF [1, 2]. Ras signaling is constitutively activated in a large fraction of human cancers [3]. Activating mutations of the three major Ras isoforms (H, K and N) were found in more than 33% of all human cancers [4-6].

Ras signaling is a major junction of various signaling pathways. For this reason, Ras and its effectors serve as important targets for therapeutic intervention. In order for Ras to be functional, it is modified by an addition of a farnesyl lipid group that allows its attachment to the membrane. A synthetic Ras inhibitor, S-*trans, trans-*farnesylthiosalicylic acid (FTS; also known as Salirasib), which structurally resembles the carboxy-terminal farnesylcysteine group common to all Ras proteins, was developed [7]. FTS acts as a functional Ras antagonist in cells, affecting Ras-membrane interactions by dislodging the protein from its anchorage domains, facilitating its degradation and, thus, reducing cellular active Ras content [8, 9]. FTS has been shown to inhibit the growth of H-Ras, K-Ras and N-Ras transformed rodent fibroblasts *in vitro* and to inhibit the anchorage-independent growth of several cancer cell lines [10-13].

Autophagy, a process of self-digestion of cellular constituents, regulates the balance between protein synthesis and protein degradation [14, 15]. Autophagy is important for normal growth, and might be defective in disease [16, 17]. It permits the elimination of damaged organelles in the cell, and allows recycling of free amino acids and nutrients in the case of nutrient deprivation or other insults. For this reason, it plays a crucial role as a cell-survival mechanism under stress conditions, such as absence of nutrients and is essential for normal cell growth and survival [18].

The formation of autophagosomes, autophagic vesicles, is controlled by several Atg proteins [17]. Atg8 proteins, also known as MAP-LC3 (in human), are associated with the autophagosomal membrane, and can serve as a marker for autophagy [14]. Additional protein which serves as an autophagy marker is the p62/SQSTM1, a scaffold protein that binds LC3 and ubiquitinated protein aggregates, and is degraded during autophagy [19].

Involvement of autophagy was documented in some forms of cancer, including hepatoma, pancreatic and breast carcinomas [17]. Chloroquine is a known drug, commonly used to prevent malaria [20]. It was demonstrated that chloroquine could inhibit autophagy by blocking lysosomal acidification and, consequently, autophagosome degradation [21]. Since chloroquine inhibits autophagosome degradation, it is expected that LC3-II levels will increase following chloroquine treatment, due to the lack of LC3 degradation.

Recent studies suggest inhibition of autophagy as a new strategy for cancer therapy [21, 22]. These studies have demonstrated that some cancers depend on autophagy for survival during external stresses, such as hypoxia, chemotherapy or radiotherapy [23]. Other studies suggest a possible involvement of Ras and autophagy in cancer cell transformation [24, 25] and addiction [26]. In the present study we examined the impact chloroquine and FTS treatments have on cell viability of two cancer cell lines which express mutant K-Ras (Panc-1 and HCT-116). In agreement with our previous report [27], we demonstrate that FTS alone induces autophagy and cell growth inhibition in these two cell lines. However, following the combined treatment, inhibition of FTS-induced autophagy by chloroquine, synergistically promoted cell growth inhibition, inhibited anchorage independent growth and enhanced caspase-dependent apoptotic cell death. These results demonstrate a mechanism by which the combined treatment of FTS and chloroquine induces cell death. Thus, the combined treatment may have a better anti-cancer effect than each drug alone.

## RESULTS

Recently, we have found that Ras inhibition by FTS can induce autophagy, which partially protects cells from FTS treatment [27]. However, the mechanism by which FTS-induced autophagy protects cells and the impact of a combined treatment with chloroquine (an autophagy inhibitor) and FTS were unknown. In the present study, we examined whether inhibition of autophagy by chloroquine will potentiate the effect of FTS on cell growth inhibition and cell death. First, we examined whether FTS induces autophagy in Panc-1 (human pancreatic cancer) and HCT-116 (human colon cancer) cell lines, both expressing mutant constitutively active K-Ras [28, 29]. In order to determine autophagy, LC3-II and p62 proteins were used as autophagy markers. Cells were treated with the indicated FTS concentrations for the indicated time periods. As shown in Figure S1A and B, the levels of LC3-II increased in a dose-dependent manner, indicating that FTS has induced autophagy in these two cell lines. Interestingly, the levels of p62 decreased only at a short time (18 h) following treatment. At longer incubation periods, p62 levels significantly increased. These results suggest that p62, which is needed for continuation of the autophagic process, may be synthesized de-novo at later stages of autophagy. Therefore, we examined whether the increase in p62 levels is due to a *de-novo* synthesis of the protein. Cells were treated with FTS in the presence or in the absence of cycloheximide (a protein synthesis inhibitor) and the levels of p62 were determined. As shown in Figure S1C, in the presence of cycloheximide, FTS had no effect on p62 levels, indicating that the increase in p62 is caused by protein synthesis. These results also suggest that the increased levels of p62 are secondary to the decrease observed shortly following FTS treatment. Interestingly, cycloheximide also causes a decrease in LC3 levels, indicating that synthesis of LC3 proteins also occurs.

**Figure 1:**
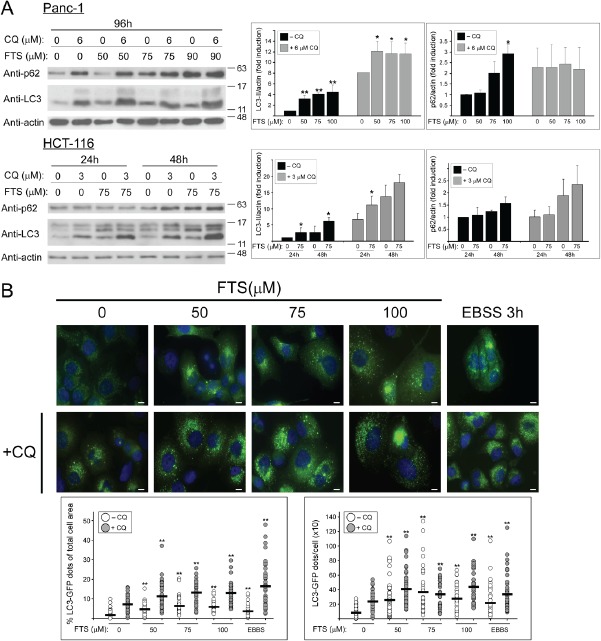
The effect of chloroquine and FTS treatments on autophagy (A) Panc-1 and HCT-116 cells were treated with FTS at the indicated concentrations, with or without chloroquine (CQ, 3 and 6 µM) for the indicated times, and then subjected to immunoblot analysis using anti-LC3 and anti-p62 antibodies. *Left panel*s, representative blots. *Right panel*s, densitometric analysis of the results is presented as fold induction over the control untreated cells (means ± S.D, n=4; *, p < 0.05 and **, p<0.01). (B) Panc-1 cells stably expressing LC3-GFP were treated with or without 7.5 µM chloroquine in the absence or presence of FTS at the indicated concentrations, for 48 h. As a control, cells were incubated with EBSS for 3 h. The cells were fixed with 4% paraformaldehyde and nuclei were stained with bisdenzimide (Hoecsht 33258). Following fixation and staining, the cells were photographed using Olympus motorized inverted research microscope Model IX81 (60×magnifcation; scale bars, 10 micrometer). *Upper panel*, representative images are shown. *Lower Panel*, autophagy was quantified by calculating the percentage of LC3-GFP dots relative to the total cell area and by counting the number LC3 dots per cell using the ImageJ software. Each dot represents a single cell (horizontal black bar: average; 40-70 cells were analyzed per treatment; **, p < 0.01).

Having demonstrated that FTS induced autophagy in these cell lines, we next examined the effect of chloroquine on LC3-II levels. Chloroquine inhibits autophagosome fusion with the lysosome, thus causing an increase in the levels of LC3-II [21]. Cells were incubated with the indicated FTS concentrations in the absence or in the presence of chloroquine. As shown in Figure 1A, chloroquine treatment, which blocks lysosomal acidification, dramatically increased the levels of LC3-II. Treatment with FTS alone has also increased LC3-II levels due to the enhancement of autophagy induction (Figure 1) and autophagic flux [27]. The combined treatment significantly enhanced LC3-II levels compared to each treatment alone. To further examine the effect of FTS and chloroquine treatment on autophagy, we have used Panc-1 cells stably expressing GFP-LC3 and followed the generation of LC3 puncta following treatments. As a positive control, EBSS treatment was used (Figure 1B). As shown, FTS treatment increased GFP-LC3-puncta. Likewise, chloroquine treatment significantly enhanced the generation of GFP-LC3 puncta. However, the combined treatment with FTS and chloroquine further enhanced autophagosomes accumulation, as judged by the increase in punctate staining. Taken together, the results indicate that chloroquine enhances FTS-induced LC3-II accumulation.

**Figure 2:**
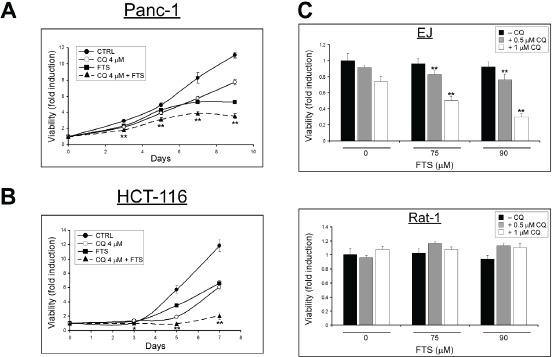
Chloroquine enhances FTS-induced cell growth inhibition (A) Panc-1 and (B) HCT-116 cells were treated with 60 or 50 µM FTS respectively, with or without 4 µM chloroquine (CQ), for the indicated times. Cell viability was assessed at different time points using the methylene blue staining assay. (C) EJ and Rat-1 cells were treated for 4 days with FTS, with or without chloroquine for the indicated concentrations. The cells were then tested for cell viability using the methylene blue staining assay. Results are presented as fold induction over the control untreated cells, and are the mean ± S.D (n=6; *, p < 0.05 and **, p<0.01 compared to each treatment alone).

Next, we examined whether FTS treatment, with or without chloroquine, affects cell viability. Panc-1 and HCT-116 cells were treated with or without FTS (60 or 50 µM respectively), in the absence or in the presence of chloroquine (4 µM), for the indicated time periods. Cell viability was determined using the methylene blue staining assay. As demonstrated in Figure 2A and 2B, FTS treatment significantly inhibited cell growth. However, the effect of FTS was significantly more pronounced in the presence of chloroquine. These results suggest that although FTS inhibits cell growth, the autophagy induced by the drug may partially protect cells, whereas inhibition of autophagy by chloroquine may increase cells sensitivity to FTS. In order to examine the contribution of active Ras to the effect of the combined treatment on cell viability, we have used Rat-1 fibroblasts and Rat-1 transfected with H-Ras (12V), known as EJ cells. As shown in Figure 2C, Ras transformed, EJ cells responded to FTS and chloroquine treatments by cell growth inhibition. In contrast, Rat-1 cells grown under the same conditions were not affected. Thus, cells expressing activated Ras are more sensitive to the treatment.

**Figure 3:**
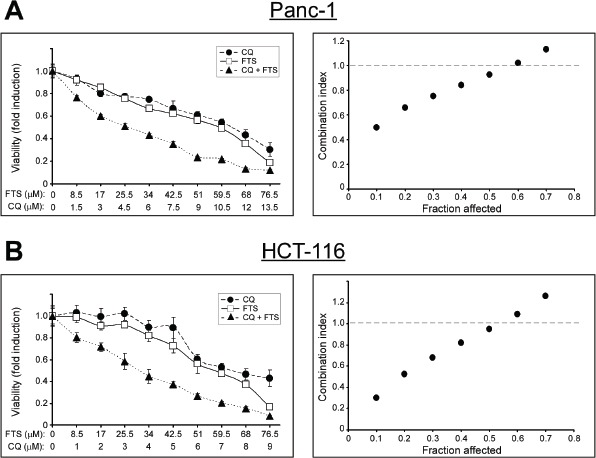
Analysis of the synergy between FTS and chloroquine (A) Panc-1 and (B) HCT-116 cells were treated for 10 and 5 days, respectively, using increasing concentrations of FTS and chloroquine (CQ), either alone or at fixed ratio (5.67:1 for Panc-1 and 8.5:1 for HCT-116 cells). *Left panels*, cell viability was tested using the methylene blue staining assay. Results are presented as fold induction over the control untreated cells, and are the mean ± S.D (n=6). *Right panels*, the combination index was calculated as described in Materials and Methods and is plotted vs. affected fraction

To further characterize the nature of the combined. FTS and chloroquine treatment (additive or synergistic) cell viability following the combined treatment at various drugs concentrations in a fixed ratio was determined (Figure 3). The data were used to calculate ‘CI’ plots to assess whether drug combination is synergistic or additive [30]. As shown, the combined treatment exhibited synergistic effect over a wide range of low concentrations in both cell lines tested (CI<1). Nonetheless, at high drugs concentrations the combined treatment was additive (CI=1).

**Figure 4:**
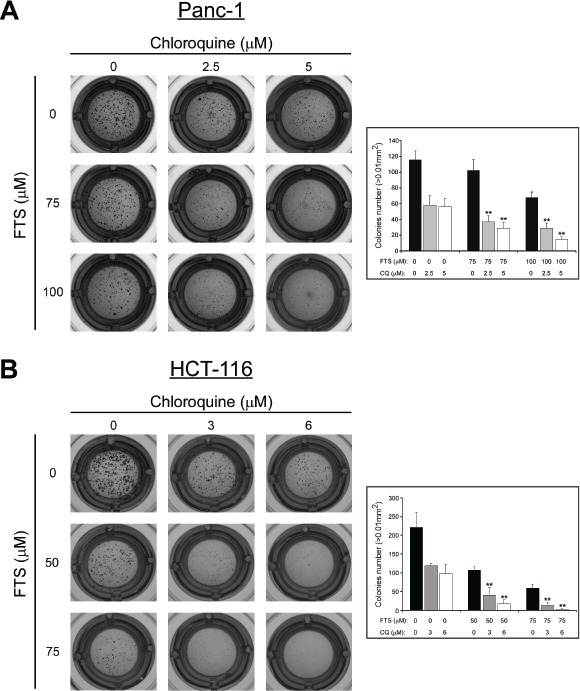
Chloroquine enhances FTS-induced inhibition of anchorage-independent growth (A) Panc-1 cells (2,700 cells/ well) and (B) HCT-116 cells (5,000 cells/well) were grown in soft agar for 13 or 11 days, respectively, in the presence of FTS, with or without chloroquine (CQ), at the indicated concentrations. Colonies were then stained as described in Materials and Methods. *Left panels*, photomicrographs of typical wells. *Right panel*s, number of colonies (>0.01 mm^2^) is presented for each treatment as mean ± SD (n=6; **, *p* < 0.01 compared to each treatment alone).

Next, we examined whether the combined treatment with FTS and chloroquine affects anchorage-independent growth. For this aim, we employed the soft agar assay. Cells were plated in soft agar and maintained in culture for 11-13 days, as indicated, before quantifying the number and size of colonies able to grow in an anchorage-independent manner. Results of typical experiments are shown in Figure 4. Under these conditions, the combined treatment induced a significantly lower number of colonies compared to each of the drugs alone, in both cell lines tested. Thus, the combined treatment appears to inhibit cell transformation.

**Figure 5:**
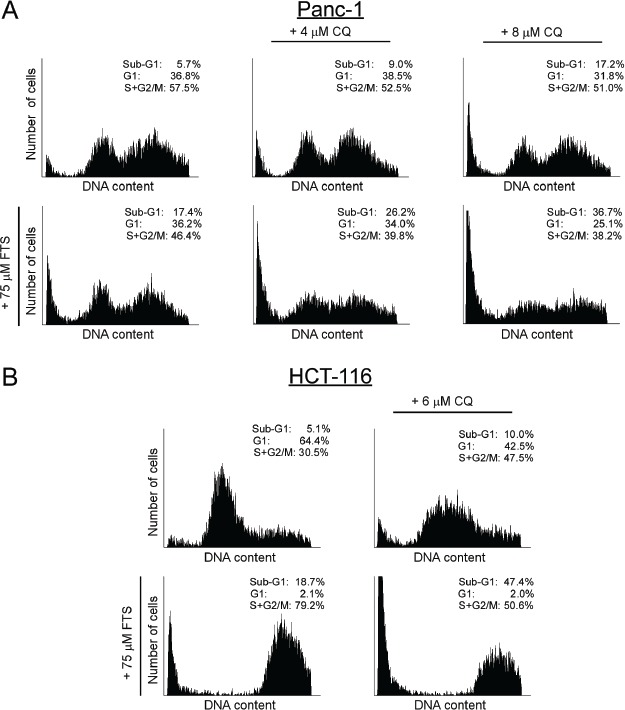
Chloroquine enhances FTS-induced increase in sub-G1 population (A) Panc-1 and (B) HCT-116 cells were treated with FTS, in the presence or absence of chloroquine (CQ) at the indicated concentrations, for 9 and 5 days, respectively. The cells were then harvested and analyzed for their DNA content by flow cytometry. The percentage of cells at various cell cycle stages is indicated.

**Figure 6:**
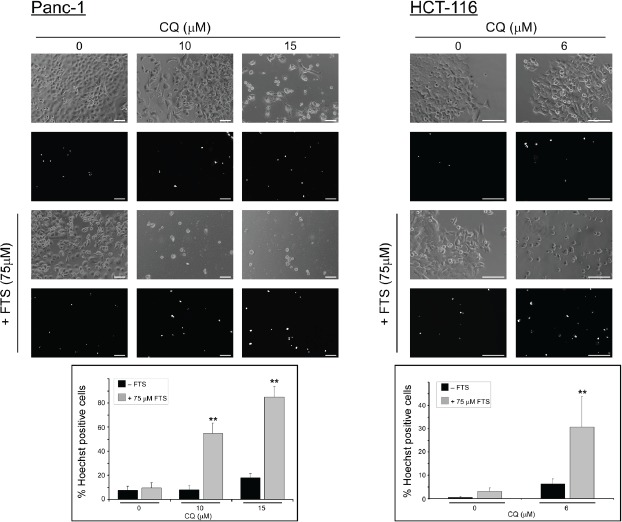
Chloroquine enhances FTS-induced cell death Panc-1 and HCT-116 cells were treated with 75 µM FTS, with or without chloroquine, at the indicated concentrations for 7 or 6 days, respectively. The cells were stained with the fluorescent DNA dye bisbenzimide (Hoechst 33258, 1 µg/ml) to assess the number of dying cells. Following staining, the cells were photographed using Olympus motorized inverted research microscope Model IX81 (20×magnifcation for HCT-116 cells and 10×magnifcation for Panc-1 cells ; scale bars, 100 micrometer). *Upper panel*, representative images. *Lower panel*, percentage of dying cells was estimated by counting the number of Hoechst-positive cells compared to the number of total cells in each field (7-10 fields for each treatment, 100-200 cells per field). Results are presented as mean ± S.D (**, p < 0.01, compared to each treatment alone).

Next, the combined treatment was tested for the induction of cell death. Two methods for detection of cell death were implimented: flow cytometry and Hoecsht dye exclusion assay. The results presented in Figure 5 and Figure 6 demonstrate that chloroquine enhances FTS-induced cell death in both cell lines, as evident by the increase in sub-G1 population (Figure 5) and by the high percentage of Hoecsht-positive cells (Figure 6). Hence, our findings clearly demonstrate that in these cells FTS induces autophagy, which partially protects cells from FTS-induced cell death and cell growth inhibition, while autophagy inhibition by chloroquine promotes FTS-mediated cell death.

**Figure 7:**
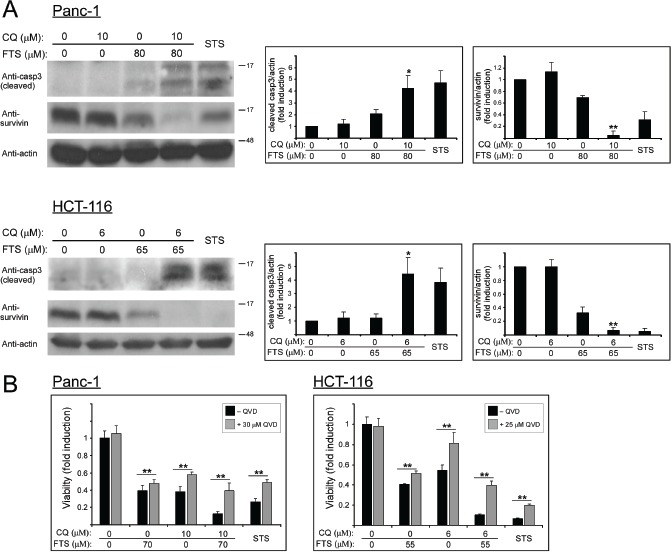
Chloroquine enhances FTS-induced apoptosis (A) Panc-1 and HCT-116 cells were treated with FTS with or without chloroquine at the indicated concentrations for 3 days, and then subjected to immunoblot analysis using anti-caspase 3 and anti-survivin antibodies. As positive control, cells were treated with staurosporine (STS) at 200 nM (Panc-1) and 150 nM (HCT-116). *Left panel*s, representative blots. *Right panel*s, densitometric analysis of the results is presented as fold induction over the control untreated cells (means ± S.D, n=3; *, p < 0.05 and **, p<0.01 compared to each treatment alone). (B) Panc-1 and HCT-116 cells were treated with FTS, chloroquine or both, with or without QVD-OPH at the indicated concentrations for 5 days. As positive control, cells were treated with staurosporine at 200 nM (Panc-1) and 150nM (HCT-116). Cell viability was assessed using the methylene blue staining assay. Results are presented as fold induction over the control untreated cells, and are the mean ± S.D (n=6; *, p < 0.05 and **, p<0.01).

**Figure 8:**
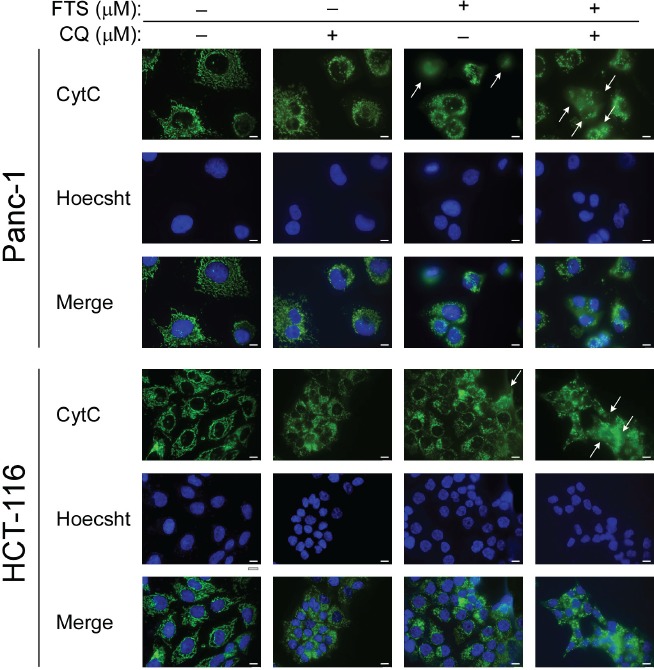
Effect of FTS and chloroquine combination on cytochrome c release Panc-1 and HCT-116 cells were treated with FTS (75 and 63 µM, respectively), with or without chloroquine (10 and 6 µM, respectively) for 48 h. The cells were fixed with 4% paraformaldehyde and immunostained with anti-cytochrome c (CytC) antibody followed by Alexa Fluor 488-labeled secondary antibody. Nuclei were stained with bisdenzimide (Hoecsht 33258). Following fixation and staining, the cells were photographed using Olympus motorized inverted research microscope Model IX81 (60×magnifcation; scale bars, 10 micrometer). Cells exhibiting evidence of cytochrome c release are indicated with arrows.

To further characterize the mechanism of cell death induced by the combined treatment, we have used two typical apoptosis markers: caspase 3 cleavage and survivin levels. As shown in Figure 7A, the combined treatment significantly enhanced caspase 3 cleavage and reduced survivin levels. We then used the broad spectrum caspase inhibitor QVD-OPH, which reduced the cell growth inhibition induced by the combined treatment of FTS and chloroquine (Figure 7B). Additional apoptotic parameter, which indicates changes in mitochondrial outer membrane permeability (MOMP), was implemented. During early stages of apoptosis, cytochrome *c* is often released from the mitochondria. We wanted to examine whether this phenomenon occurred in cells treated with FTS and chloroquine. The cellular distribution of cytochrome *c* in the treated cells was examined by immunofluorescence staining with anti-cytochrome *c* antibodies. As shown in Figure 8, healthy control cells exhibited a scattered, punctuated cytochrome *c* staining pattern, typical of the subcellular distribution of mitochondria, whereas the cells co-treated with FTS and chloroquine exhibited a diffuse cytochrome *c* staining pattern, suggestive of release of cytochrome *c* from the mitochondria. Consequently, it appears that the combined treatment of chloroquine and FTS induces caspase dependent apoptotic cell death in HCT-116 and Panc-1 cells.

## DISCUSSION

Ras signaling is a major junction of various signaling pathways, which control cell viability. Several studies have shown that oncogenic Ras can elevate the pro-survival activity of autophagy [22, 31, 32]. However, other studies had demonstrated that Ras-driven autophagy might be a part of a pro-death mechanism. In particular, that H-Ras-induced autophagy contributes to caspase-independent cell death [33]. Ras was also found to upregulate beclin 1 and a knockdown of the key autophagy genes *BECN1*, *ATG5* or *ATG7* was shown to reduce oncogenic Ras-mediated cell death [33, 34]. On the other hand, several studies showed that active Ras could inhibit autophagy [35, 36]. Recently, we have demonstrated that FTS (a Ras inhibitor) promotes autophagy in treated cells [27]. In the present study we demonstrate that FTS induces autophagy also in Panc-1 and HCT-116 cancer cell lines, both expressing mutant constitutively active K-Ras. Therefore, inhibition of Ras by FTS promotes autophagy in several cancer cells.

Autophagy is an evolutionarily conserved process, which functions as a tumor suppression mechanism by removing damaged organelles/proteins and limiting cell growth and genomic instability [37]. Most studies demonstrate that autophagy plays a role in cell survival. However, cell death, resulting from uncontrolled excessive autophagy, was also documented [38, 39]. Evidence also exist that autophagy has a role not only in tumor suppression, but also as a process involved in oncogenesis [25]. Several studies implicated a complex interplay between autophagy and cancer. Previously, we have demonstrated that autophagy induced by FTS partially protects cells from death [27]. These results motivated us to further study the effect of FTS treatment in combination with autophagy inhibitor on cancer cells viability. Since chloroquine has been safely used for many years to treat malaria, it was chosen for the present study as an autophagy inhibitor [20]. It was also shown to have favorable effects as a novel antitumor drug in several studies [22, 40, 41]. Indeed, our results indicate that inhibition of autophagy by chloroquine resulted in enhanced cell growth inhibition and even enhanced cell death in response to FTS treatment. The inhibitory effect of the combined treatment was synergistic, suggesting that each of the inhibitors may affect a distinct pathway. Furthermore, we also found that H-Ras-transformed cells were more sensitive to the combined treatment compared to naïve cells, which means that Ras-transformed cells respond better to the treatment. Moreover, the combined treatment seems to inhibit anchorage independent growth of cancer cells. It also appears that the combined treatment enhances caspase-dependent apoptotic cell death as judged by typical apoptotic assays. Accordingly, we suggest that in addition to the ability of FTS to inhibit cell growth, it also induces pro-survival autophagy and pro-death pathways. Inhibition of autophagy by chloroquine strengthens the cell death pathways induced by FTS. In fact, our study suggests that the combined inhibition of Ras by FTS and autophagy by chloroquine has a greater potential as anti-cancer treatment compared to each drug alone.

## MATERIALS AND METHODS

### Materials and buffers

Antibodies were obtained from the following sources: monoclonal mouse anti-actin (MP Biomedicals, Santa Ana, CA; 691001), monoclonal mouse anti-cytochrome c (BD Biosciences, San Jose, CA; 556432) polyclonal rabbit anti-caspase 3 (Santa Cruz Biotechnology, Dallas, TX; sc-7148), polyclonal rabbit anti-survivin (Santa Cruz Biotechnology; sc-10811) polyclonal rabbit anti-LC3B (Sigma-Aldrich, St. Louis, MO; L7543) and polyclonal rabbit anti p62 (MBL Intenational, Woburn, MA; PM045). Reagents are as follows: Salirasib (FTS, S-trans, trans-farnesylthiosalicylic acid), chloroquine (CQ, C6628), cycloheximide (CHX, 01810) and staurosporine (STS, S5921) were from Sigma-Aldrich. QVD-OPH was from R&D Systems (Minneapolis, MN; OPH001).

### Cell lines

The human cancer cell lines Panc-1 and HCT-116 were grown in DMEM (Gibco, Carlsbad, CA) and McCoy's 5A modified medium (Sigma-Aldrich), respectively. Rat-1 fbroblast cells and H-Ras-transformed Rat-1 cells (EJ cells) were grown in DMEM. All media were supplemented with antibiotics and 10% heat-inactivated fetal bovine serum (FBS; Hyclone, Thermo Scientific, Waltham, MA). Cells were incubated at 37°C in 5% CO2 in air, and the medium was changed every 3–4 days. When 70% confluent, cells were passaged in trypsin/ disodium ethylenediaminetetraacetic acid (Biological Industries, Beit-Haemek, Israel). One day before treatment the cells were plated at ~50% confluence in medium supplemented with 5% heat-inactivated fetal bovine serum (10% for EJ and Rat-1 cells). Concentrations for FTS treatments (and control treatments with 0.1% DMSO) are indicated for each experiment, as well as the duration of treatment, when relevant.

### Stable transfection

Panc-1 cells were stably transfected with the Lipofectamine 2000 reagent (Invitrogen, Carlsbad, CA) according to the manufacturer's instructions. Stable clones expressing LC3-GFP were selected and cultured with 400 µg/ml geneticin (G-418, Calbiochem, San Diego, CA).

### Assays of cell survival and cell death

Cells were plated in medium supplemented with 5% FBS and treated as indicated for the different experiments. Cell numbers were determined by the methylene blue assay. For this purpose, the cells were fixed with 4% formaldehyde in phosphate-buffered saline for 2 hours, then washed once with 0.1 M boric acid (pH 8.5) and incubated with the DNA-binding dye methylene blue (1% in boric acid) for 20 minutes at room temperature. The cells were then washed three times with distilled water and lysed with 0.1 M HCl. Absorbance was measured with a Tecan Spectrafluor Plus spectrophotometer (Mannedorf, Switzerland) at 595 nm. Cell viability was calculated as the ratio of absorbance in treated cultures to that in untreated control cultures.

To estimate the number of dead cells, live cultures were incubated for 10 minutes with 1 μg/ml of the fluorescent DNA dye bisbenzimide (Hoechst 33258; Sigma). After staining, the cells were photographed with an Olympus motorized inverted research microscope Model IX81. The percentage of dead cells was estimated by calculating the number of Hoechst-stained nuclei relative to the total cell number in each field.

### Cell cycle analysis

Cells were plated in medium supplemented with 5% FBS and treated as indicated. After treatment, the cells were trypsinized, washed once with PBS, and fixed in cold methanol for 15 minutes. Fixed cells were washed once with PBS and incubated at 4°C for 30 minutes. RNase A (0.05 mg/ml) and propidium iodide (0.05 mg/ ml) were added and the stained cells were analyzed in a fluorescence-activated cell sorter (FACScan; Becton Dickinson, Franklin Lakes, NJ) within 30 minutes. Percentage of cells at different stages of the cell cycle was determined using the WinMDI 2.9 program.

### Lysate preparation and Western Blot analyses

After the indicated treatment, cells were lysed in solubilization buffer (50 mM HEPES pH 7.5, 150 mM NaCl, 10% glycerol, 1% Triton X-100, 1 mM EDTA pH 8, 1 mM EGTA pH 8, 1.5 mM MgCl2, 200 µM Na3VO4, 150 nM aprotinin, 1 µM leupeptin and 500 µM 4-(2-aminoethyl) benzenesulfonyl fluoride hydrochloride). Lysates were cleared by centrifugation, and a boiling gel sample buffer was added. Lysates were resolved by sodium dodecyl sulfate polyacrylamide gel electrophoresis through 10%-12.5% polyacrylamide gels, and were electrophoretically transferred to nitrocellulose membranes. Membranes were blocked for 1 hour in TBST buffer (0.05 M Tris-HCl pH 7.5, 0.15 M NaCl, and 0.1% Tween 20) containing 6% milk, and then blotted with primary antibodies for 2 hours. Secondary antibody, linked to horseradish peroxidase, was then added for 1 hour. Immunoreactive bands were detected with the enhanced chemiluminescence reagent. Densitometric analysis of the results was performed using the ImageJ program.

### Anchorage-independent growth assay

Noble agars (2% and 0.6%) were prepared in double-distilled water and autoclaved. The 2% agar was melted and mixed with medium (concentrated ×2 with 20% FCS), and the mixture (50 µl) was placed in 96-well plates to provide the base agar (at a final concentration of 1%). The cells were suspended in medium (concentrated ×2 with 20% FCS, mixed with 0.6% agar) and 50 µl of the mixture were plated on the base agar. 100 µl of medium (×1, 10% FCS) containing the indicated treatments (concentrated×2) were added to the wells. The plates were incubated for the 10-14 days at 37°C. Colonies were then stained with 25 µl 3-(4,5- dimethylthiazol-2-yl)-2,5-diphenyltetrazolium bromide (5 mg/ml) and photomicrographed. The number of colonies per well (>0.01 mm2) was determined using the ImagePro software.

### Analysis of the cytotoxic effect of FTS in combination with chloroquine

The effect of drug combination was calculated according to the median effect principle, described by Chou and Talalay (1984). First, we constructed the dose– response curves for the cytotoxic effects of FTS and chloroquine alone, and in combination, in Panc-1 and HCT-116 cells using the methylene blue staining assay. The data was used to determine the ‘combination index’ (CI), using the equation: CI = (D)1/(Dx)1+(D)2/(Dx)2, where (D)1 and (D)2 are the combinations doses that kill x% of cells, and (Dx)1 and (Dx)2 are the doses of each drug alone that kill x% of cells. If CI<1, then synergism is indicated.

### Statistical analysis

All experiments were performed at least three times. Results are presented as means ± SD. Differences between means were assessed by the 1-tailed Student's t-test. Significance was assigned at p < 0.05.

## Supplemental Figure


