# Supplementary material for: Chloroquine synergizes with FTS to enhance cell growth inhibition and cell death
Source: Oncotarget. 2013 Nov 20;5(1):173–84. doi: 10.18632/oncotarget.1500 (PMC3960199; doi:10.18632/oncotarget.1500)
Supplement: Supplementary file 1 [file oncotarget-05-0173-s001.pdf]

Chloroquine synergizes with FTS to enhance cell growth inhibition and cell death - Schmukler et al

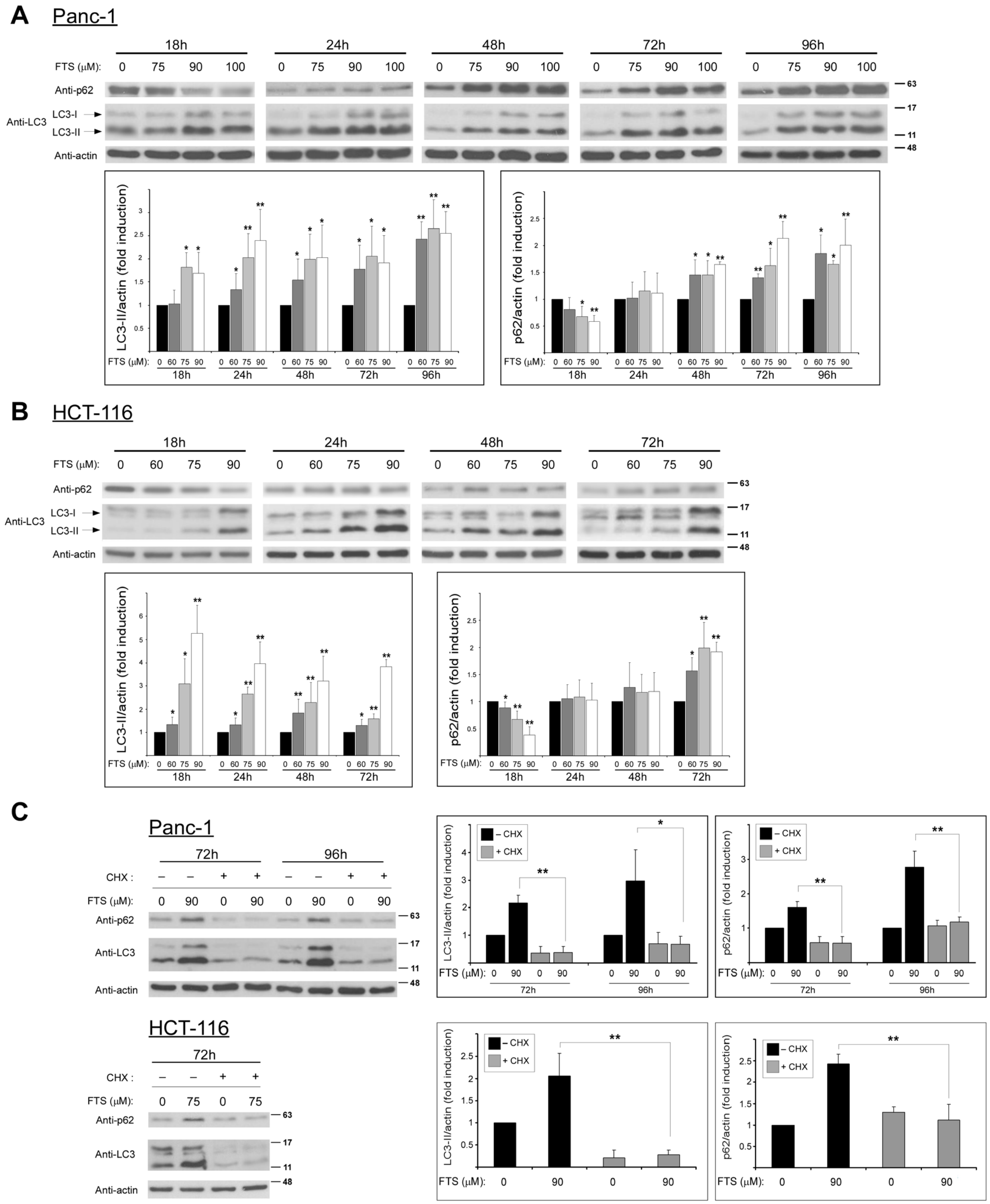

Figure S1: Time course of FTS-induced LC3-II accumulation and p62 degradation. (A) Panc-1 and (B) HCT-116 cells were treated with FTS for the indicated concentrations and time periods, and then subjected to immunoblot analysis using anti-LC3 and anti-p62 antibodies. Upper panels, representative blots. Lower panels, densitometric analysis of the results is presented as fold induction over the control untreated cells. (C) Panc-1 and HCT-116 cells were treated with FTS at the indicated concentrations, with or without cycloheximide (CHX, 50μg/ml) for the indicated times, and then subjected to immunoblot analysis using anti-LC3 and anti-p62 antibodies. Left panels, representative blots. Right panels, densitometric analysis of the results is presented as fold induction over the control untreated cells (means ± S.D, n=4; *, p < 0.05 and **, p<0.01).
